# How many papillomavirus species can go undetected in papilloma lesions?

**DOI:** 10.1038/srep36480

**Published:** 2016-11-03

**Authors:** Cíntia Daudt, Flavio R. C. da Silva, André F. Streck, Matheus N. Weber, Fabiana Q. Mayer, Samuel P. Cibulski, Cláudio W. Canal

**Affiliations:** 1 Laboratório de Virologia, Faculdade de Veterinária, Universidade Federal do Rio Grande do Sul (Av. Bento Gonçalves, 9090, Prédio 42.602, CEP 91540-000, Porto Alegre, Brazil); 2 Centro de Ciências Biológicas e da Natureza, Universidade Federal do Acre (Campus Universitário, BR 364, Km 04 - Distrito Industrial- CEP: 69920-900, Rio Branco, Brazil); 3 Universidade de Caxias do Sul (Rua Francisco Getúlio Vargas, 1130, 95070-560, Caxias do Sul, Brazil); 4 Laboratório de Biologia Molecular, Instituto de Pesquisas Veterinárias Desidério Finamor (IPVDF), Fundação Estadual de Pesquisa Agropecuária (Estrada do Conde, 6000, CEP 92990-000, Eldorado do Sul, Rio Grande do Sul, Brazil)

## Abstract

A co-infection comprising to at least seven papillomavirus (PV) types was detected by next generation sequencing (NGS) of randomly primed rolling circle amplification (RCA) products of a bovine (*Bos taurus*) papilloma lesion from the Brazilian Amazon region. Six putative new PV types that could not be detected by commonly used PCR protocols were identified. Their overall L1 nucleotide identities were less than 90% compared to described PV species and types. L1 nucleotide BLAST sequence hits showed that each new type was related to *Beta, Gamma*, *Dyokappa*, *Dyoeta*, and *Xipapillomavirus*, as well as two likely new unclassified genera. Our results show that the employment of NGS is relevant to the detection and characterization of distantly related PV and is of major importance in co-infection studies. This knowledge will help us understand the biology and pathogenesis of PV, as well as contribute to disease control. Moreover, we can also conclude that there are many unknown circulating PVs.

Papillomaviruses (PVs) are circular double-stranded DNA viruses containing approximately 8,000 base pairs (bp)^1^,^2^. They belong to the *Papillomaviridae* family and these complex viruses can infect a wide range of amniotes^1^,^2^,^3^. This large family is composed of viruses phylogenetically assigned to 39 genera with several species, types, subtypes and variants^1^,^2^. The entire genome must be sequenced for considering new PV types and the L1 open reading frame (ORF) have to differ by more than 10% in comparison to the closest PV types. PV species share between 71% and 89% nucleotide identity within the complete L1 ORF. A PV genus is defined when the similarities are larger than 60% in the L1 ORF. When this difference is between 2% and 10% or less than 2% a subtype or variant occurs, respectively^1^,^2^.

Human PV (HPV) encompasses more than 200 types that are fully sequenced, characterized and cataloged, in contrast to the low number of *Bos taurus papillomavirus* (BPV), which comprises only 15 types (http://pave.niaid.nih.gov). PVs are usually characterized by PCR amplicon sequencing, which is performed using degenerate primer pairs that amplify a relatively conserved L1 gene region from all known PV types and species^4^. This technique has allowed the identification of divers putative new PVs types in humans and other animals, including BPV types in cattle herds from distinct continents worldwide^5^,^6^,^7^,^8^.

Although PV detection and characterization in animal species are still poorly studied^3^,^9^, primers directed to the PV genera have been commonly used and have shown an increase in the specificity of detection and characterization of PV DNA compared to general primers^10^,^11^,^12^. Nearly two hundred human PVs (HPVs) have been recognized and PV co-infections have been reported using numerous molecular techniques that detect many distinct HPVs genotypes^13^,^14^,^15^,^16^. Several generic PV primer systems have been developed for the detection of HPV types rather than BPV types^17^. Additionally, there is much less research of cattle in this field, reflected by the vague BPV types reported and the few cases of BPV co-infection reported^18^,^19^,^20^.

Multiply primed rolling circle amplification (RCA) of circular genomes followed by classic sequencing enabled the discovery of novel animal PVs^21^. Although this study did not show evidence of co-infections, the RCA followed by NGS has enabled the detection of HPV co-infections^22^. Moreover, the extraordinary diversity of PV types that infect the animal skin combined with the low numbers of PV types detected by degenerate primers^23^ indicate that these techniques allow the discovery of only closely related PVs from known genera. Furthermore, PVs could be cultivated only in a sophisticated and arduous raft cell culture system, thereby hampering whole genome analysis due to the lack of necessary adequate amount of purified genomic viral DNA^24^.

Therefore, an efficient method for amplification and sequencing is essential for improving the identification of PV species that are not detected by current methods mainly in animal researches. The knowledge of PV types is important for epidemiological studies of viral variants in different PV-affected species and to determine the full picture of genetic diversity. Such information will help clarify the biological relationship between distinct viruses with respect to both pathogenesis and treatment. Along these lines, randomly primed RCA followed by NGS was performed to investigate the complete genetic diversity of PVs present in a papilloma lesion.

## Results

### NGS from RCA products reveals distinct BPV genomes

NGS from RCA products of one papilloma lesion enabled the amplification of seven full-length PV genomes. The contigs associated with PV were assembled from 113,616 high-quality reads (Table 1). The seven contigs were named BPV13 BR/14RO and putative BPV BR/14RO-16 to BPV BR/14RO-21. Chimeric forms were not detected using the RDP4 software. Primer alignment with the Geneious software (version 9) (http://www.geneious.com)^25^ found mismatches in all sequences at the 3’ end of the FAP59 and MY09 primers (Supplementary Figure 1). A low number of reads probably corresponding to the sequence 14RO_12 (GenBank accession number KP701419) was also identified in the sample. Since the genome was not complete it was not further analyzed.

**Figure 1:**
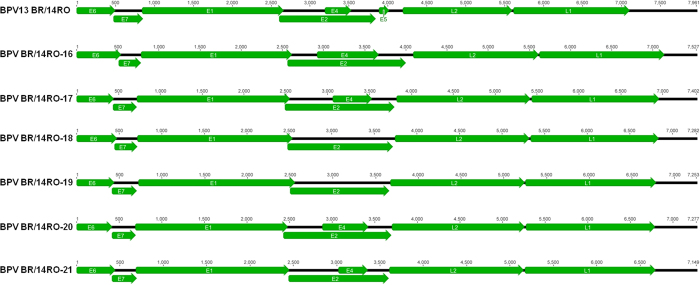
PV genomes found in this study and their archetypical organization. The first nucleotide in the ORF6 was assigned a number 1 in the sequences. All putative new papillomavirus genomes (BPV BR/RO-16 to BPV BR/RO-21) were predicted to contain six to eight ORFs, coding for early (E6, E7, E1, E2, E4 and E5) and late (L1 and L2) proteins.

The genomes are 7,149 to 7,961 Kb in length and display the archetypal organization of PVs (Fig. 1). The first nucleotide in E6 was assigned the number 1 in the sequences. All putative new viruses (BPV BR/14RO-16 to BPV BR/14RO-21) were predicted to contain six (BPV BR/14RO-18 and 19) to seven ORFs (BPV BR/14RO-16, BPV BR/14RO-17, BPV BR/14RO-20 and BPV BR/14RO-21), coding for early (E6, E7, E1, E2, and E4) and late (L1 and L2) proteins (Fig. 1). The BPV13 variant sequence showed the same characteristics as the reference genome^17^. The overall L1 nucleotide identities of the putative new types were less than 90% in comparison to other PV species and types^1^.

### Phylogenetic inferences

**Figure 2:**
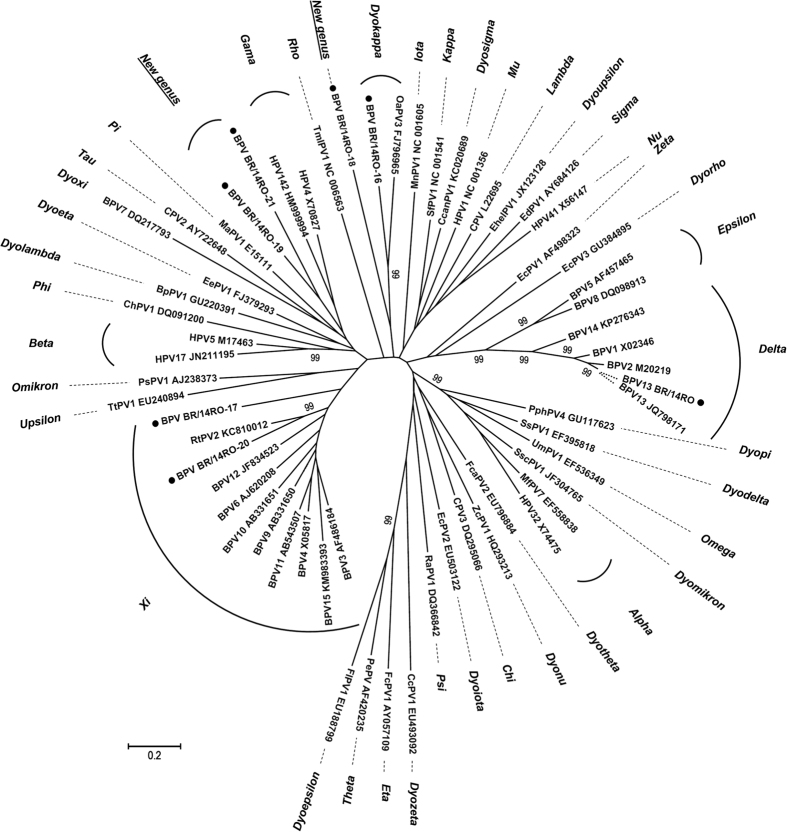
Phylogenetic tree of the papillomaviruses based on complete sequences of the L1 ORF. Bootstrap repetitions (higher than 99%) are indicated above the main branches. Samples belonging of this study with representatives of other PV genera were included on the analysis. A total of 61 PV types of different species and genera were analyzed. Accession numbers for the sequences are included and abbreviations are used according to PaVE. Putative new types and genera are indicated with black dots.

Phylogenetic inferences showed that the six new PV genomes clustered with three known and two unknown PV genera (Fig. 2). Their nucleotide and amino acid identities to the closest related PVs are summarized in Table 2. L1 identities to the most closely related PVs with corresponding GenBank accession numbers following phylogenetic analysis are summarized in Table 2. Putative BPV BR/14RO-16 clustered with members of *Dyokappapapillomavirus* and was most closely related to *Ovis aries* PV3 (OvPV3) (62% sequence identity). Putative BPV BR/14RO-17 was most closely related to BPV3 (62%), and putative BPV BR/14RO-20 was most closely related to RtPV2 (*Rangifer tarandus* PV2) (74% sequence identity), both members of the *Xipapillomavirus* genus. Putative BPV BR/14RO-18was most closely related to members of the *Xi* and *Dyokappapapillomavirus* genera and likely represents a new genus. Putative BPV BR/14RO-19 and BPV BR/14RO-21 constituted a distinct cluster and were most closely related to PVs belonging to *Gamma, Xi* and *Dyoetapapillomavirus*. Both are probable representatives of a new genus in the *Papillomaviridae* family. The BPV13 BR/14RO and the putative new BPV BR/14RO-16 to BPVBR/14RO-21 sequences were deposited in GenBank under accession numbers KU519390 to KU519396.

## Discussion

Remarkable efforts have been made to identify human PVs using numerous clinical arrays that can detect dozens of distinct HPV genotypes in the same sample^13^,^14^ using several generic PV primer systems^17^. These efforts reflect over than 200 HPV genomes that are fully sequenced, characterized and cataloged (PaVE). In comparison to HPV, only 15 BPV types have been detected and fully sequenced thus far (PaVE). This scenario is probably pictured because of the lower efforts in BPV studies when compared to HPV due clinical relevance and funding applied. Co-infections comprising HPV are commonly reported in young or immunodepressed women^26^,^27^,^28^. On the other hand, BPV co-infections comprising up to six known PV types based on multiplex BPV genotyping assay^20^ or specific primers^18^,^29^ are rarely reported. Although the majority of BPV types and putative new types have been characterized using generic or genus-specific primers^8^,^12^,^17^,^30^, such protocols have important limitations because they allow the discovery of only closely related PVs but rarely detect mixed infections. In the present study, the combination of RCA and NGS allowed the detection of at least seven BPVs co-infecting the same lesion, including six putative new BPV types.

An isothermal protocol that uses ϕ29 DNA polymerase to amplify complete PV genomes was previously developed^31^. Following the amplification of the genome, there is the need to analyze the DNA using restriction enzymes, cloning, and sequencing which is labor-intensive and time-consuming. Additionally, multiply primed RCA and primer-walking for entire genome sequencing already enabled the discovery of novel PVs^21^. Moreover, there is no evidence of co-infection. One possibility is that the specific degenerate primer pairs used in these studies selected a virus population with higher affinity to primer binding than unknown viruses that may be present in the same sample. This could lead to an underestimation of the detection of other viruses in mixed infections and even a failure to detect distant phylogenetic PVs.

A RCA followed by NGS approach was applied to analyze a sample from which have been previously found a putative new BPV type^8^. The method enabled the detection of seven PVs with six being uncharacterized so far. The randomly primed RCA followed by NGS offers the possibility to amplify and detect any circular DNA that is present in a sample without specificity, thus allowing a great overview of unknown PVs. This technique could magnify the sensitivity of all PVs present in one sample and allow the understanding of the natural history of infection by different PV types. This approach is meaningful once there is more evidence suggesting that cervical infections caused by some HPV types may also depend on the existence of other HPV types^13^, thereby suggesting a synergistic pattern. NGS present some restrictions as limiting capability to find mutations like single nucleotide polymorphisms (SNP), insertions and deletions in regions of lower coverage^32^,^33^. To minimize the possible base calling errors, in the present study, only high quality reads (Q ≥ 30) were used for *de novo* assembly. Also, although this Illumina platform displays some underestimation in AT-rich and CG- rich regions^33^ all putative new types and the BPV13 described in this study presented a GC content considered normal when compared to other PV family members.

The present method enables the detection of a large number of putative new types suggesting the existence of many other BPV types that may have been underestimated thus far. Such a massive PV co-infection indicates that these mammals can harbor genetically diverse PVs similar to humans. Additionally, the Amazon region ecosystem harbors one of the largest global biodiversities and it is quite propitious for the emergence of novel strains. However, there is a need for deeper investigations on this issue by applying this method in all PV affected animals, including other cattle herds worldwide and humans.

We have shown that the enrichment method together with the Illumina NGS platform works for a range of PV genera detection such as *Dyokappa*, *Xi* and *Gammapapillomavirus*. Moreover, the identity of three new types showed inter-genera localization, and these types probably compose two new genera in the *Papillomaviridae* family. These findings indicate a high number of undetected PVs ignored in the usual assays. Therefore, it is essential to use unbiased methods for the discovery of highly divergent novel viruses as has been done with numerous human and animal agents^34^.

Most of the xipapillomaviruses present E5 or E8 gene in the E6 genomic position, including BPV4, BPV9, BPV10, BPV11 and BPV15 (GenBank accession no. X05817, AB331650, AB331651, AB543507, and KM983393, respectively) that encode E5, and BPV3, BPV6 and BPV12 (GenBank accession no. AF486184, AJ620208, and JF834523, respectively) that encode E8. The novel xipapillomaviruses detected in the present study present E6 in the E6 genomic position as well as RtPV2 that clustered in the same terminal node that BR/14RO-20. BR/14RO-17 formed a separated branch within *Xipapillomavirus* and probably is a novel species.

In conclusion, the combination of two relatively simple and fast methods already developed to amplify and genotype PV genomes proved to be very effective in the detection of known and unknown PV viruses using small amounts of DNA derived from one PV lesion. Furthermore, viral genomes can be largely reconstituted by currently available *de novo* assembly algorithms. The presence of multiple PV types and variants in the same lesion will allow the development of new studies regarding the roles of these different viruses in the biology and pathogenesis of the diseases in which they are involved.

## Material and Methods

### Ethics Statement

Lesions were collected by veterinarians to prepare autogenous vaccines and all efforts were made to minimize animal suffering. All procedures were performed in compliance with the European Convention for the Protection of Vertebrate Animals Used for Experimental and Other Scientific Purposes (European Treaty Series—No. 170 revised 2005) and the procedures of the Brazilian College of Animal Experimentation (COBEA). It must be highlighted that this project was approved by Universidade Federal do Rio Grande do Sul Animal Ethics Committee (number 28460) and we had the owner’s permission to access the animals for the purposes of this study.

### Sample processing, rolling circle amplification, and Illumina sequencing

Biopsy material was obtained from a bovine of the Brazilian Amazonian region, diagnosed with epidermal papillomatosis. The lesion was removed using scalpels after local anesthesia (performed with 2% lidocaine, Bravet, Brazil). One putative new PV type was previously detected when a L1 fragment was sequenced using FAP primers^8^. To obtain the complete genome sequence of this putative new type, the tissue was processed and genomic DNA was extracted as described previously^8^. To amplify the full PV genomes, randomly-primed rolling circle amplification (RCA) was performed essentially as described by Rector *et al.*^31^ using 100 ng of purified DNA from the biopsy specimen. The amplicons were analyzed by agarose gel electrophoresis stained with Blue Green Loading Dye I (LGC, Brazil) and examined under UV light with the Molecular Imaging Software Gel Logic (Kodak, USA).

Following RCA, DNA was purified using a Genomic DNA Clean & Concentrator (Zymo Research). The quality and quantity of the DNA were assessed using a Nanodrop spectrophotometer (Thermo Scientific) and a Qubit fluorometer (Invitrogen). DNA fragment libraries were further prepared with one ng of purified RCA DNA using a Nextera XT DNA sample preparation kit and sequenced using an Illumina MiSeq instrument (2 × 150 paired-end reads with the Illumina v2 reagent kit).

### Genome assemblies and sequence analyses

The paired-end sequence reads were assembled into contigs using SPAdes 3.5^35^ and compared to sequences in the GenBank nucleotide and protein databases using BLASTn/BLASTx. Geneious software version 9^25^ was used for open reading frame (ORF) and conserved domain predictions as well as genome annotation. Motif Scan (http://myhits.isb-sib.ch/cgi-bin/motifscan) was used to confirm the conserved domain prediction pointed by Geneious software version 9^25^.

Similarities searches were performed using local sequence alignments BLAST^36^. Global sequence alignments were accomplished to determine sequence identities with MUSCLE software^37^. Representative sequences within genera and sequences with the highest identities to the sequences from the present study that are available in GenBank were retrieved from the NCBI homepage (http://www.ncbi.nlm.nih.gov/) for phylogenetic analysis. Altogether, the dataset consisted of 45 sequences of the L1 gene. The multiple sequence alignments was performed through the MUSCLE software^37^.

The phylogeny was reconstructed with a maximum likelihood method using the MEGA6 software^38^. These analyses were performed using the GTR substitution model, and the algorithm was modeled with a gamma distribution (shape parameter = 5). The nucleotide substitution model was defined by the tool “find best DNA/Protein model (ML)” of MEGA6 software^38^. Statistical support was provided by 1,000 non-parametric bootstrap analyses.

RDP4 software^39^, using the RDP^40^, GENECONV^41^, BOOTSCAN^42^, MAXCHI^43^, CHIMAERA^44^, SISCAN^45^ and 3 SEQ^46^ methods using default settings were used to look for the presence of chimeric genomes that can arise during the building of contigs. Recombination was considered credible in sequences only if they were detected by more than three methods having significant *P* values coupled with strong phylogenetic support for recombination. To verify any mismatches that could make difficult the annealing of the viruses detected with the degenerate primer regions, all generated sequences were aligned with primers FAP59/64^4^ and MY09/11^47^ using ClustalX software^38^.

## Additional Information

**How to cite this article**: Daudt, C. *et al.* How many papillomavirus species can go undetected in papilloma lesions? *Sci. Rep.* **6**, 36480; doi: 10.1038/srep36480 (2016).

**Publisher’s note:** Springer Nature remains neutral with regard to jurisdictional claims in published maps and institutional affiliations.

## Supplementary Material

Supplementary Information

## Figures and Tables

**Table 1:** Overall genome coverage and GC-content of the seven contig papillomavirus sequences recovered from a papillomatous lesion.

	GenBank acession no.	Read count*	Mean genome coverage#	GC-content (%)
**BPV13 BR/14RO**	KU519390	1645	31	45.1
**BPV BR/14RO-16**	KU519391	650	12	44.5
**BPV BR/14RO-17**	KU519392	1366	27	42.4
**BPV BR/14RO-18**	KU519393	578	12	41.0
**BPV BR/14RO-19**	KU519394	774	16	42.7
**BPV BR/14RO-20**	KU519395	378	8	44.6
**BPV BR/14RO-21**	KU519396	630	13	47.7

^*^Number of reads mapped to the reference.

^#^Average number of times each base was sequenced.

**Table 2:** L1 identities to most closely related PVs with corresponding GenBank accession numbers following phylogenetic analysis.

PNT#	Identity (%)	PV type	PV species	PV genera	GenBank acession
BPV BR/14RO-16	62	OaPV3	*Dyokappapapillomavirus 1*	*Dyokappa*	FJ796965
	55	BPV3	*Xipapillomavirus 1*	*Xi*	AF486184
	55	BPV6	*Xipapillomavirus 1*	*Xi*	AJ620208
	55	BPV9	*Xipapillomavirus 1*	*Xi*	AB331650
	55	BPV11	*Xipapillomavirus 1*	*Xi*	AB543507
BPV BR/14RO-17	62	BPV3	*Xipapillomavirus 1*	*Xi*	AF486184
	61	BPV4	*Xipapillomavirus 1*	*Xi*	X05817
	61	BPV6	*Xipapillomavirus 1*	*Xi*	AJ620208
	61	RtPV2	*Xipapillomavirus 3*	*Xi*	KC810012
BPV BR/14RO-18	54	RtPV2	*Xipapillomavirus 3*	*Xi*	KC810012
	53	OaPV3	*Dyokappapapillomavirus 1*	*Dyokappa*	FJ796965
	51	BPV BR/14RO-16	*	*Dyokappa*	KU519391
BPV BR/14RO-19	66	BPV BR/14RO-21	*	*	KU519396
	60	HPV4	*Gammapapillomavirus 10*	*Gamma*	X70827
	60	EePV1	*Dyoetapapillomavirus 1*	*Dyoeta*	FJ379293
	59	MaPV1	*Pipapillomavirus 1*	*Pi*	E15111
	59	HPV142	*Gammapapillomavirus 10*	*Gamma*	HM999994
BPV BR/14RO-20	74	RtPV2	*Xipapillomavirus 3*	*Xi*	KC810012
	68	BPV12	*Xipapillomavirus 2*	*Xi*	JF834523
	67	BPV9	*Xipapillomavirus 1*	*Xi*	AB331650
BPV BR/14RO-21	66	BPV BR/14RO-19	*	*	KU519394
	58	HPV142	*Gammapapillomavirus 10*	*Gamma*	HM999994
	58	BPV6	*Xipapillomavirus 1*	*Xi*	AJ620208

^*^Genomes not assigned to PV species or genera.

^#^Putative new types (PNT) are designated as BPV BR/14RO-16 to BPV BR/14RO-21.
