# Supplementary material for: How many papillomavirus species can go undetected in papilloma lesions?
Source: Sci Rep. 2016 Nov 3;6:36480. doi: 10.1038/srep36480 (PMC5093584; doi:10.1038/srep36480)
Supplement: Supplementary Information [file srep36480-s1.doc]

**How many papillomavirus species can go undetected in papilloma lesions?**

Cíntia Daudt, Flavio R. C. da Silva, André F. Streck, Matheus N. Weber, Fabiana Q. Mayer, Samuel P. Cibulski, Cláudio W. Canal

**Supplementary Figure 1. Nucleotide alignment plots of primer-annealing sites in BPV sequences.** (A) FAP59 and FAP64 primers. (B) MY11 and MY09 primers. BPV1 (GenBank accession no. NC_001522) was used as reference sequence. Primer positions are given in the 5' to 3' direction. Alignment were generated by MUSCLE with default settings and the figure is drawn in Geneious software (version 9) ^24^. "-" symbols represent a amplification product of ~430 and 410 bp in FAP and MY primers, respectively.
